# Editorial: Novel biomarkers for clinical and molecular stratification of organ involvement in rheumatic diseases

**DOI:** 10.3389/fmed.2023.1274950

**Published:** 2023-08-28

**Authors:** Xiaolin Sun, Miao Pan

**Affiliations:** ^1^Department of Rheumatology and Immunology, Peking University People's Hospital, Beijing, China; ^2^Division of Pathology and Laboratory Medicine, Children's National Hospital, Washington, DC, United States; ^3^Departments of Pathology and Pediatrics, The George Washington University School of Medicine and Health Sciences, Washington, DC, United States

**Keywords:** rheumatic disease, biomarker, organ involvement, molecular stratification, precision medicine

Multiple organ involvements develop in most of the leading rheumatic diseases including rheumatoid arthritis, systemic lupus erythematosus, systemic vasculitis, etc. While progress has been made in advancing diagnosis and treatment strategies, the elusive goal of early prediction, accurate diagnosis, and precise treatment endures. This challenge persists due to the absence of robust biomarkers and practical predictive models that can reliably guide clinicians in navigating the complex and diverse manifestations of these conditions. The integration of genomics, proteomics, and high-throughput screening, holds great potential for unearthing these elusive biomarkers. In this Research Topic of Frontier in Medicine, multiple studies deciphered the intricate molecular signatures associated with rheumatic disease and organ involvement patterns, which pave the way for the development of diagnostic tests that enable early detection and prediction. Additionally, the data generated from these technologies can be harnessed to construct predictive models that account for the complex interplay of variables contributing to disease progression.

Systemic lupus erythematosus (SLE) is a typical systemic rheumatic disease affecting most organs of the body. However, there is still an unmet need of clinically useful biomarkers that can predict organ involvement for personalized treatment. Fenton and Pedersen reviewed current research progress to identify advanced methods and possible biomarkers to be utilized in the diagnosis, disease monitoring, and prediction of treatment response in SLE. The authors proposed to look for biomarkers and methods that have specificity and sensitivity for distinct organ involvement across different diseases. Combined strategies with biomarkers, multi-omics methods and novel clinical measurements such as molecular imaging might be the best strategy to explore the precision medicine of SLE (Fenton and Pedersen). In a mini review, Tsuchida et al. summarized the abnormal expression and pathogenic roles of autotaxin in patients with SLE. Autotaxin is produced by plasmacytoid dendritic cells and is associated with type I interferons, which may become a potential biomarker in SLE (Tsuchida et al.).

Hydroxychloroquine (HCQ) is another immunosuppressant commonly used in treatment of autoimmune disease such as SLE. A small fraction of patients taking HCQ may develop acute generalized exanthematous pustulosis (AGEP) as an adverse response. Luo et al. explored the clinical features and associated gene expression of AGEP induced in HCQ treated patients with rheumatic diseases including SLE, RA, or pSS, and showed that HCQ-induced AGEP might have a longer latency period and regression time than AGEP induced by other drugs. CARD14 gene mutations might contribute to the molecular basis of AGEP (Luo et al.).

Sjögren's syndrome (SS) is a systemic autoimmune disease characterized by the immune system attacking and damaging the exocrine glands, leading to a reduction in their secretory function. The existing diagnostic methodology for SS is both invasive and lacking in efficiency, necessitating the exploration of more advanced and effective approaches. In a systematic investigation by Kamounah et al., the proteomics and miRNA-expression profile were established and analyzed. It is found that upregulation of cystatin A, β2M, α-enolase, actin, E-FABP, N-GAL, Ig-κ light-chain and C3 as well as downregulation of PRPs, CA6, α-amylase, histatins, PIP, cystatin S, and cystatin SN in patients with SS. The discriminatory performance of an anti-SSA/Ro combined with TRIM29 showed a higher sensitivity than anti-SSA/Ro positivity alone.

Rheumatoid arthritis (RA) is a chronic joint destructive autoimmune disease leading to systemic involvement and disabling. First-line treatment for RA is methotrexate (MTX), but 20–40% of RA patients do not respond to MTX (Brynedal et al.). It is therefore important to identify the variable biological effect of MTX between responders and non-responders. By employing a multi-omics approach that combines the strengths of flow cytometry, RNA sequencing, and multiplex protein quantification, Brynedal et al. performed a deep molecular and cellular phenotyping of peripheral blood cells in RA patients treated with MTX. This integrated strategy enabled them to elucidate the differences between responders and non-responders and the results might be helpful to explore the mechanism of non-responsiveness to MTX in RA treatment.

With bioinformatical analysis, Sun et al. established a lncRNA–miRNA–mRNA network in circulating exosomes (cirexos) to identify potential biomarkers for systemic sclerosis (SSc). Differentially expressed mRNAs (DEmRNAs) and lncRNAs (DElncRNAs) in SSc cirexos were screened and the ENST00000313807-hsa-miR-29a-3p-COL1A1 network in plasma cirexos could perform as a combined biomarker for the clinical diagnosis and treatment of SSc.

Systemic vasculitis are composed of a series of heterogeneous autoimmune diseases characterized by blood vessel inflammation potentially involving multiple organs and systems. Umezawa et al. reported that leucine-richα-2 glycoprotein (LRG), could be a novel biomarker for CRP-negative patients to indicate disease activity.

Liu et al. investigated the correlation between *FGA* gene polymorphisms and coronary artery lesion in Kawasaki disease (KD). Their study showed that *FGA* gene polymorphisms affected coronary artery lesion in children with KD (Liu et al.). Lung involvement is one of the most severe organ damages in patients with myeloperoxidase (MPO)-anti-neutrophil cytoplasmic antibody (ANCA)-associated vasculitis (MPO-AAV). Chen et al. exploited the image features of lung involvement to predict the therapeutic response and facilitate decision making in MPO-AAV treatment. They developed and validated a radiomics nomogram model to predict treatment resistance of Chinese MPO-AAV patients based on low-dose multiple slices computed tomography (MSCT) of the involved lung with cohorts from two centers, and this model might work as a non-invasive tool for predicting treatment response and improve individualizing treatment decisions (Chen et al.).

Overall, this Research Topic focus on *Novel biomarkers for clinical and molecular stratification of organ involvement in rheumatic diseases*. Over the past decades, numerous advances have been made in multiple omic approaches, which enable us to screen clinical samples and combine multi-layer data to exploit useful biomarkers. Some of the innovations might become part of the routine clinical practice in the future.

## Author contributions

XS: Writing—original draft, Writing—review and editing. MP: Writing—original draft, Writing—review and editing.

